# Requirements for reflection in the critical care environment

**DOI:** 10.4102/curationis.v38i1.1202

**Published:** 2015-03-09

**Authors:** Celia J. Filmalter, Tanya Heyns

**Affiliations:** 1 Department of Nursing Science, University of Pretoria, South Africa

## Abstract

**Background:**

Reflection is recognised as an important method for practice development. The importance of reflection is well documented in the literature, but the requirements for reflection remain unclear.

**Objectives:**

To explore and describe the requirements for reflection in the critical care environment as viewed by educators of qualified critical care nurses.

**Method:**

A focus group interview was conducted to explore and describe the views of educators of qualified critical care nurses regarding requirements for reflection in the critical care environment.

**Results:**

The themes that emerged from the focus group were buy-in from stakeholders – management, facilitators and critical care nurses, and the need to create an environment where reflection can occur.

**Conclusion:**

Critical care nurses should be allowed time to reflect on their practice and be supported by peers as well as a facilitator in a non-intimidating way to promote emancipatory practice development.

## Introduction

Reflection is a pivotal skill for all critical care nurses who have to care for patients in an ever-changing medical environment. The origins of reflection can be traced back to John Dewey, a philosopher of education, who explained reflection as an intentional, active progression of learning (Nelson 2012). Reflection forms part of the experiential learning cycle for the purpose of assisting practitioners to make sense of an experience and to seek alternatives and improved actions for practice (Passarelli & Kolb 2011). A modern scholar of reflection, Johns (2013) recognised the value of reflection in a specific context and defines reflection as:

a window through which the practitioners can view and focus self within the context of their own lived experience in ways that enable them to confront and understand and work towards resolving the contradictions with their practice between that is desirable and actual practice. (p. 34)

Practice development applies reflection as a method to integrate work-based learning for sustained development in practice to promote the effectiveness of individuals, teams and healthcare organisations (McCormack, Manley & Titchen 2013). Nurses working in different contexts are required to develop their practice and utilise scientific evidence to guide desirable practice to improve patient outcomes.

Efforts to develop practice by educators and organisations focus on technical practice development, which is task orientated and uses top-down approaches to learning (McCormack *et al*. 2013). There is limited literature on the approaches used in South Africa to develop practice; however, in a study conducted in the United Kingdom it was found that if practice development was conducted by using traditional teaching strategies, the nurses became passive recipients of information only (Williams 2010). The resources spent using traditional teaching strategies for practice development make no significant change in how nurses practise in their work context (Duff, Gardner & Osborne 2014; Williams 2010). In order for practice development to bring about sustainable change and positive outcomes for both patients and the nursing profession, McCormack *et al*. (2013) advocate the use of emancipatory practice development.

Emancipatory practice development focuses on improving outcomes and self-understanding of nurses and assisting them to become critics of their social and educational situations, as well as their work context and workplace culture (Manley, McCormack & Wilson 2008:268). Emancipatory practice development is divided into three phases: enlightenment, empowerment and emancipation. Wilson and McCormack (2006) describe enlightenment as the phase where nurses are made aware of the changes that are required; through empowerment nurses gain information that enables them to act on the changes identified. Emancipation is achieved when enlightened and empowered nurses take action to reform their practice in the context of their work (Boomer & McCormack 2010).

For nurses to become enlightened about their unarticulated assumptions about themselves and their practice, reflection can be used to improve their practice. Reflection is a method to assist nurses to take personal responsibility for actions and practice development for sustainable change (Boomer & McCormack 2010).

### Problem statement

Shifting qualified critical care nurses from being passive recipients of information to being active participants in their practice development requires them to engage in reflection. Reflection is, however, a taught skill and requires a necessary shift in the education and training of qualified critical care nurses (Berterö 2010; McCormack *et al*. 2013). Evidence of reflection being used for practice development is scarce.

Black and Plowright (2010) are of the opinion that vagueness and the popularisation of reflection, as well as perplexing theories and inconsistent study results on the topic of reflection, have presented problems for teaching, training and use of reflection in practice. Boomer and McCormack (2010) confirm that reflection is a learned skill that requires development.

The views of educators involved in the practice development of qualified critical care nurses, that were responsible for introducing and facilitating reflection in the practice setting, were explored to reveal requirements for realising reflection in the critical care environment.

## Research method and design

An explorative, descriptive, qualitative design was used to gain insight into the requirements for reflection. Educators from private and public healthcare facilities involved in the practice development of qualified critical care nurses were invited to participate in a focus group discussion (FGD). Participants for the FGD were identified through the use of snowball sampling. Seven participants were identified during the sampling process and invited to the FGD, which was conducted at a venue which was central for all the participants. Informed consent was obtained from participants and their right to withdraw from the FGD at any time was reiterated before commencement.

After permission was obtained to audio-record, the FGD began. The FGD was used to collect data through social interaction and to build upon the answers of the other participants. An independent expert in FGDs facilitated the interview, since the researcher was also involved in the practice development of qualified critical care nurses. The facilitator ensured that the interview remained focused on the topic and asked questions to clarify meanings.

The question posed to the participants during the FGD was: What are the requirements for reflection in the critical care environment?

The FGD was transcribed verbatim and studied using content analysis. The transcriptions were read and re-read to allow the researcher to become immersed in the data and sensitised to important issues. The data were analysed and coded by the researcher and an independent coder who signed a confidentiality agreement. The independent coder, with experience in coding of qualitative research, was utilised to enhance the quality of analysis of the data and in this way strengthen credibility and trustworthiness. The researcher and independent coder held a contact session in which consensus on the themes was reached.

## Ethical considerations

Written permission was obtained from the Ethics Committee of the University of Pretoria and the research board of a private hospital group. Participants were invited to an FGD a month in advance of the set date to enable them to make prior arrangements that could ensure minimal interruption of their work. Participants signed written consent prior to their participation in the data collection, and were informed of their right to withdraw at any stage. Each participant was given the opportunity to voice their view. During the transcription of data participants were assigned random names. Only the researcher had access to the true identities of the participants. Transcripts of the FGD and field notes were stored at a different location from that of the results and discussion.

## Trustworthiness

The trustworthiness of the study was ensured through the use of Lincoln and Guba's model (1985).

Credibility was ensured by referential adequacy. Data must be adequate to allow for later analysis and interpretation (Lincoln & Guba 1985). The data were audio-recorded and transcribed, ensuring that raw data could undergo further analysis and interpretation. Member checks added to the credibility (Polit & Beck 2006), as participants gave comments on the preliminary study findings.

Transferability refers to the extent to which the findings could be applied to other contexts (Lincoln & Guba 1985). The findings of this study could be applied to other contexts, as they address the requirements for realising reflective practice in the clinical setting.

Dependability entails truthfulness and reliability (Topping 2010). The use of an independent coder ensured that the data analysis was objective and accurate. Confirmability refers to the objectivity of the data (Topping 2010), and using the skills of an independent coder and an expert supervisor helped to ensure this in this study.

## Results and discussion

Healthcare organisations and patients demand critical care nurses who are innovative and use the latest knowledge in their daily practice. Reflection might provide some guidance to critical care nurses in order to meet these demands (Mann, Gordon & MacLeod 2009). Participants identified that buy-in from stakeholders and settings which are conducive to reflection are necessary for reflection to occur in the critical care environment.

### Buy-in from stakeholders

The stakeholders that were identified were organisational management, clinical facilitators and critical care nurses. The participants identified organisational management and made specific reference to the unit manager, as the unit manager who is the supervisor of critical care nurses has a direct impact on the extent to which reflection could be practised. Furthermore the unit manager could appoint a facilitator to act as a guide for reflection. Participants stated that facilitators were important stakeholders who could activate reflection that would contribute to learning. The participants agreed that: ‘… you need a facilitator to drive learning and reflection’ and ‘reflection is very difficult; not all critical care nurses know what reflection is so you have to guide them how to reflect.’

Emancipatory practice development necessitates a skilled facilitator (McCormack *et al*. 2013). The facilitator plays a pivotal role in enabling critical care nurses to engage in reflection and reflective practice. According to Johns (2013), a facilitator can create a space for critical care nurses to reflect on their practice. With facilitated reflection the critical care nurses are guided by the facilitator to internalise the knowledge from the experience and discuss how the knowledge was gained and how it could prepare them for a variety of situations in the future (Berterö 2010). However, for facilitators to be credible they too should engage in reflection and reflective practice to enhance their facilitation skills. According to Tomlin, Weatherston and Pavkov (2014:74) facilitators should be ‘compassionate, tolerant/non-judgemental, self-reflective, reliable and predictable’ in order to be effective. A facilitator that portrays the above characteristics could be capable of building a trusting and supportive relationship with the critical care nurse.

The participants were also concerned about the reaction of critical care nurses towards reflection. The following quotations summarise this concern:

‘… it is with a type of personality … I found it is very difficult for critical care nurses to reflect and to … um … admit that they did something wrong and could've done something better. They don't respond positively …’

‘… when it comes to critical care nurses they are not like students and you can anticipate resistance (toward reflection) …’

Nelson (2012) and Berterö (2010) claim that most nurses are aware and acknowledge the importance of reflection. For reflection to assist critical care nurses to continuously enhance their practice, they should be open, collaborative, non-defensive, realistic and willing to ask for help (Tomlin *et al*. 2014). However, Johns (2013) is of the opinion that critical care nurses could be blunted or numbed because of the ‘unsympathetic’ working environments they are exposed to daily, and therefore might act defensively. The approach to be used by the facilitator to introduce reflection to the critical care nurses is, however, unclear and would need further investigation.

### Settings conducive to reflection

The next theme identified was that a setting should be created for reflective practice in the critical care environment. During the data collection participants made comments on the role of management to create a setting that is conducive to reflection. Boud and Hager (2011) agree that organisations and management influence practitioners’ ability to reflect by creating either a restrictive or enabling work environment. The participants agreed on the following comment:

‘… if the management structure and the unit is in such a manner that they will allow them [*critical care nurses*] to have time for reflection, then it will work, but if the environment is not conducive to it, it will not work …’

However, the use of reflection by critical care nurses is not dependent on the organisation and management alone. The setting for reflection is highly influenced by the social environment and relationships amongst critical care nurses and clinical facilitators (Gregory, Hopwood & Boud 2014). The participants perceived the relationship between the critical care nurse and the facilitator as very important, as is evident in the next two statements:

‘… if the facilitator doesn't have good relationships with the critical care nurses then reflection will not work …’

‘… your relationship with the critical care nurse is important. You cannot just come in and say: Listen here, I am now the facilitator and you will learn to reflect and you will do it like this. They [*critical care nurses*] are professionals and adults …’

Henderson *et al*. (2011) are of the opinion that a reflective partnership should be formed between the critical care nurse and clinical facilitator to enhance the practice development of both parties.

## Limitations

The findings of this study are limited by the small sample size, as this decreased the capacity to generalise the results. However, the participants were of the opinion that the results of the study were a starting-point for realising reflection in the critical care environment.

### Implications for education of critical care nurses

Educators involved in the practice development of qualified critical care nurses could enlighten the stakeholders identified in the study regarding the use and benefits of reflection. Educators should actively engage in reflection to develop their own practice. Critical care nurses should be taught the skill of reflection whilst the educators build an open, honest reflective partnership with them.

## Conclusion

This article focused on the requirements for reflection into the critical care environment, as viewed by educators of qualified critical care nurses. The ability of reflection must not be assumed but should be developed. The skill to reflect must be nurtured in a pre-established, reassuring environment, enabling critical care nurses to reflect. In order to create such an environment there should be buy-in from stakeholders to develop reflection and the creation of a setting conducive to reflection.

Should reflection not be realised in practice, critical care nurses will remain passive receivers of information and emancipatory practice and sustainable change will be hindered. Considerably more work needs to be done to determine the views and opinions of critical care nurses and management of the healthcare organisations regarding the use of reflection to improve patient outcomes in the critical care environment.
